# A novel high-throughput qPCR chip for solving co-infections in RAS farmed rainbow trout

**DOI:** 10.1038/s41598-024-65697-8

**Published:** 2024-07-22

**Authors:** Juliane Sørensen, Argelia Cuenca, Jacob Günther Schmidt, Simon Brøndgaard Madsen, Tine Moesgaard Iburg, Lone Madsen, Niccoló Vendramin

**Affiliations:** 1 National Institute of Aquatic Resources DTU Aqua, Section for Fish and Shellfish Diseases, Technical University of Denmark, Kgs. Lyngby, 2800 Denmark; 2 Højslev, 7840 Denmark

**Keywords:** Microbiology, Molecular biology

## Abstract

Recirculating aquaculture systems (RAS) have become more attractive due to reduced water consumption and effluent discharge. However, intensification of production increases the risk of introducing pathogens at farming sites. The emergence of uncultivable pathogens and RAS pathobiome diversity shifts the traditional disease paradigm from “one pathogen, one disease” to complex multiple-pathogen disease cases. *Piscine orthoreovirus* genotype 3 (PRV-3) is an excellent example, as it is capable of inducing anemia and heart pathology resembling heart and skeletal muscle inflammation under experimental conditions, and is associated with increased mortality in association with other pathogens in the field. The aim of this study was to develop a method for detection of multiple pathogens and putative pathogens, as co-infections are common in aquaculture. To do this, in the pilot study, we mapped the pathobiome of RAS-farmed rainbow trout (*Oncorhynchus mykiss*) (commercial RAS, farm A) using both standard diagnostic methods and metabarcording (16S rRNA) to investigate the gill microbiome. During this study, we observed infections with multiple pathogens, and detected two putative gill pathogens *Candidatus* Branchiomonas cysticola and *Candidatus* Piscichlamydia salmonis, both of which have been linked with complex gill disease in Atlantic salmon (*Salmo salar*). Based on the pilot study, we developed and tested a high throughput qPCR (HT-qPCR) chip targeting 22 viral and bacterial pathogens and putative pathogens, followed by a surveillance of a fish cohort in a commercial RAS farm during production (farm B). Co-infection with PRV-3 and *Ca.* B. cysticola combined with stress inducing management practices may explain the severe disease outbreak observed (37% mortality). The time course study sets the base for a future screening scheme for disease prediction and addresses limitations of the method when testing environmental DNA/RNA.

## Introduction

The global aquaculture production has grown steadily since the 1990s until today^1^. In certain geographical areas, stringent environmental restrictions implemented to minimize pollution from aquaculture plants have initiated a rapid technological development of recirculating aquaculture systems (RAS). An example of this is Denmark, where the number of fish farms decreased from five-hundred in 1990 to three-hundred in 2010, and in contrast, the total production of portion size (300 g) rainbow trout has increased over the years^1^. Despite that RAS production has the potential of being pathogen free, intensification of production implies more frequent introduction of eggs or juveniles at each site, consequently increasing the risk of introducing microbial pathogens. As a potential consequence, multiple infections may occur at fish farms, and low virulent or opportunistic pathogens may benefit from synergistic effects with other pathogens resulting in severe disease outbreaks^2^.

*Piscine orthoreovirus* genotype 3 (PRV-3) has been reported in association with disease outbreaks with high mortality and heart pathology resembling heart and skeletal muscle inflammation (HSMI) in rainbow trout (*Oncorhynchus mykiss*) in both Denmark and Norway^3–6^. Attempts to find virulence factors have so far been unsuccessful - isolates of PRV-3 associated with disease outbreaks have shown to be identical to isolates from non-disease cases as well as isolates from 1995^5^. Additionally, experimental challenges with PRV-3 report no mortality, even with environmental stressors such as low and high water temperature^4,6,7^. All this suggests that PRV-3 is not the sole cause of PRV-3 associated disease outbreaks.

Several experimental studies show increased pathology and mortality during different co-infections, and field investigations highlight that farmed fish are rarely challenged by single pathogens, but rather by multiple pathogens simultaneously: In farmed rainbow trout, co-infection with *Yersinia ruckeri* and *Pseudomonas fluorescens* was reported in three farms in Turkey^8^; PRV-3 has previously been detected together with *Flavobacterium psychrophilum*, *Renibacterium salmoninarum*, and infectious pancreatic necrosis virus (IPNV)^9^; and co-infection with IPNV and *F. psychrophilum* has been reported in rainbow trout fry, in which both pathogens were found in the same cells in some cases (endothelial cells of head kidney)^10^. An experimental challenge with *Myxobolus cerebralis* and *Tetracapsuloides bryosalmonae* reported increased mortality in co-infected groups^11^. Likewise, co-infection with *F. psychrophilum* and infectious hematopoietic necrosis virus (IHNV) showed increased mortality in co-infected fish compared to either pathogen alone^12^.

Historically, fish disease diagnostic is based on indications from clinical signs which are normally corroborated with laboratory testing for the suspected pathogen. This approach however relies on the hypothesis of one disease is the result of one pathogen, therefore overlooking potential co-infections. Furthermore, the emergence of uncultivable viral and bacterial pathogens further limit the applicability of traditional propagation on cell culture and agar plates, and thus warrant for specific molecular assay. This highlights the importance of a tool for detecting multiple pathogens simultaneously. The aim of this study was therefore to develop a molecular method for detection of multiple pathogens in aquaculture simultaneously. To determine which pathogens should be included in this tool, we conducted a pilot study mapping the pathobiome of RAS farmed rainbow trout using standard diagnostic methods (cultivation on agar, qPCR and cell culture) and 16S microbiome analysis (farm A). We subsequently designed and developed a high-throughput diagnostic qPCR (HT-qPCR) tool for simultaneous detection of 22 different pathogens affecting rainbow trout (*Oncorhynchus mykiss*) and Atlantic salmon (*Salmo salar*). To assess the new tool in the field, we followed a RAS farm (farm B) for seven months, collecting samples from clinically affected and clinically healthy fish, in order to gain on overview of the known pathogens challenging a fish batch. Additionally, production data (disease outbreaks, mortality, treatment and movement) were collected during the study.

## Results

### Pilot study, farm A: infectious agents are prevalent in RAS farmed rainbow trout

In the reported pilot study, a RAS farm (farm A) was sampled three times (June 2019, November 2019, and February 2020) in order to investigate the pathogens challenging rainbow trout farmed in Danish RAS. For this purpose, both standard diagnostic methods, gill histopathology, and 16S RNA metabarcoding of gills were employed.

**Figure 1:**
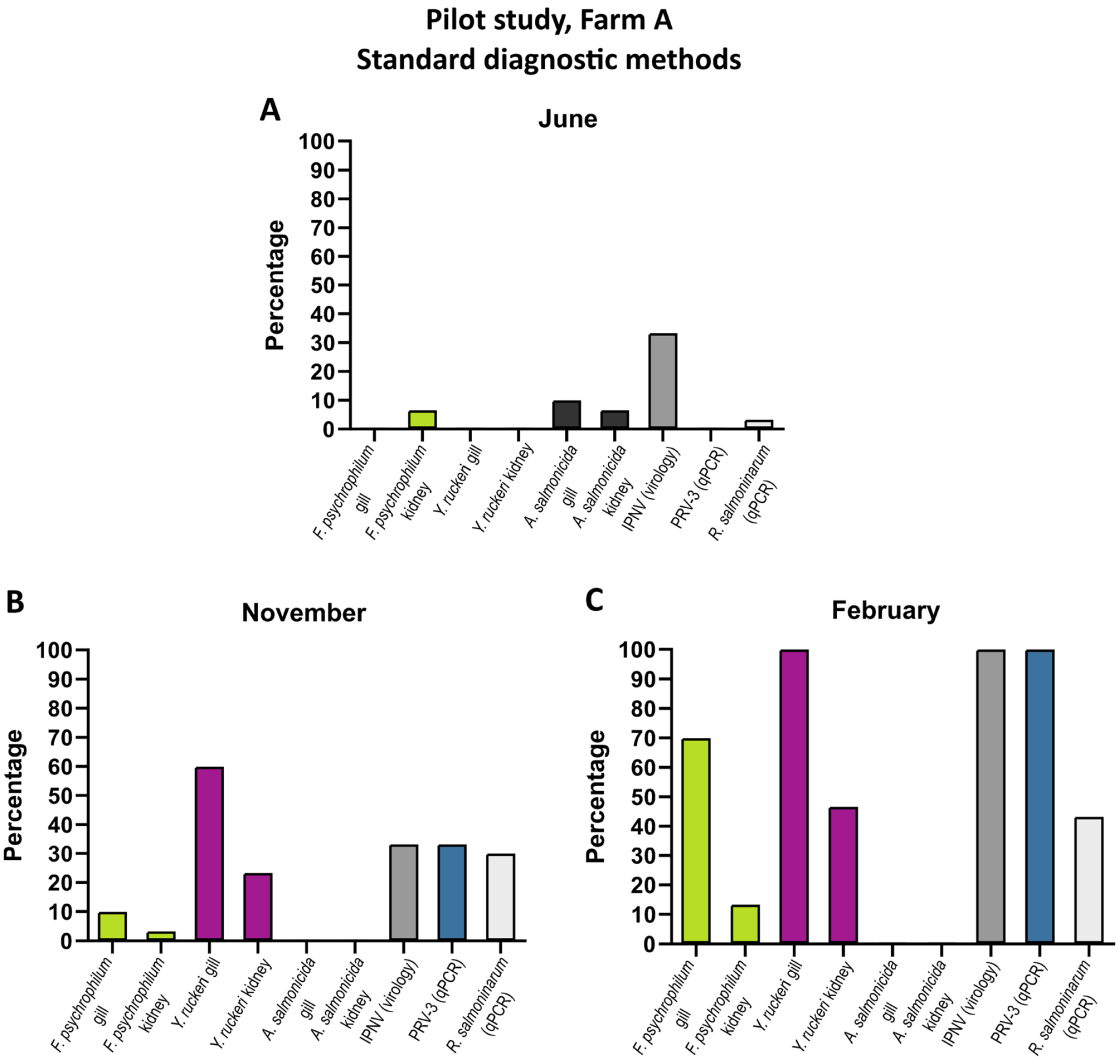
Prevalence of selected pathogens detected by standard diagnostic methods (cultivation on agar and identification on MAlDI-TOF, cell culture and PCR) for the pilot study (farm A) at three different time points: (**A**) June 2019, (**B**) November 2019, and (**C**) February 2020. The figure was generated using GraphPad Prism 10 (version 10.2.3 (403)) and Inkscape 1.2.2 (732a01da63, 2022-12-09).

This study showed that at any given sampling point, multiple pathogens were observed co-existing in farmed rainbow trout (see Fig. 1). Pathogen prevalence as well as diversity of increased over time: At the first sampling point (June 2019), few bacterial pathogens (*F. psychrophilum* and *Aeromonas salmonicida*) were detected together with IPNV. More bacterial pathogens were found at the subsequent samplings (November 2019 and February 2020). Particularly at the third sampling point (February 2020) almost all the examined fish were positive for *F. psychrophilum* and *Y. ruckeri* in gill swabs, and IPNV and PRV-3 in internal organs. *A. salmonicida* was the only pathogen which was observed at the first sampling without being detected at subsequent sampling periods.

The presence of bacterial pathogens detected by traditional diagnostics (cultivation and identification with MALDI-TOF) were corroborated by 16S rRNA gene sequencing, in which *Flavobacterium* sp., *Yersinia* sp., *Aeromonas* sp and *Renibacterium salmoninarum* were found (See Supplementary Fig. S1). However, the microbial community in the gills varied over time. Most noticeable is the presence of *Rhodoferax* sp. in February 2020, a bacterium found in stagnant waters and ponds that are enriched with nutrients^13^. The 16S analysis indicated the presence of other potentially pathogenic bacteria not yet described in Denmark, such as *Candidatus* Branchiomonas and *Candidatus* Piscichlamydia (see Supplementary Fig. S1). These putative gill pathogens^14,15^ were observed in two separate samples, *Ca.* Branchiomonas in June 2019 (0.9 and 0.1% of the reads), and *Ca.* Piscichlamydia in November 2019 (1.1 and 11.5% of the reads), although at low levels.

#### Pilot study, farm A: gill histopathology

**Figure 2:**
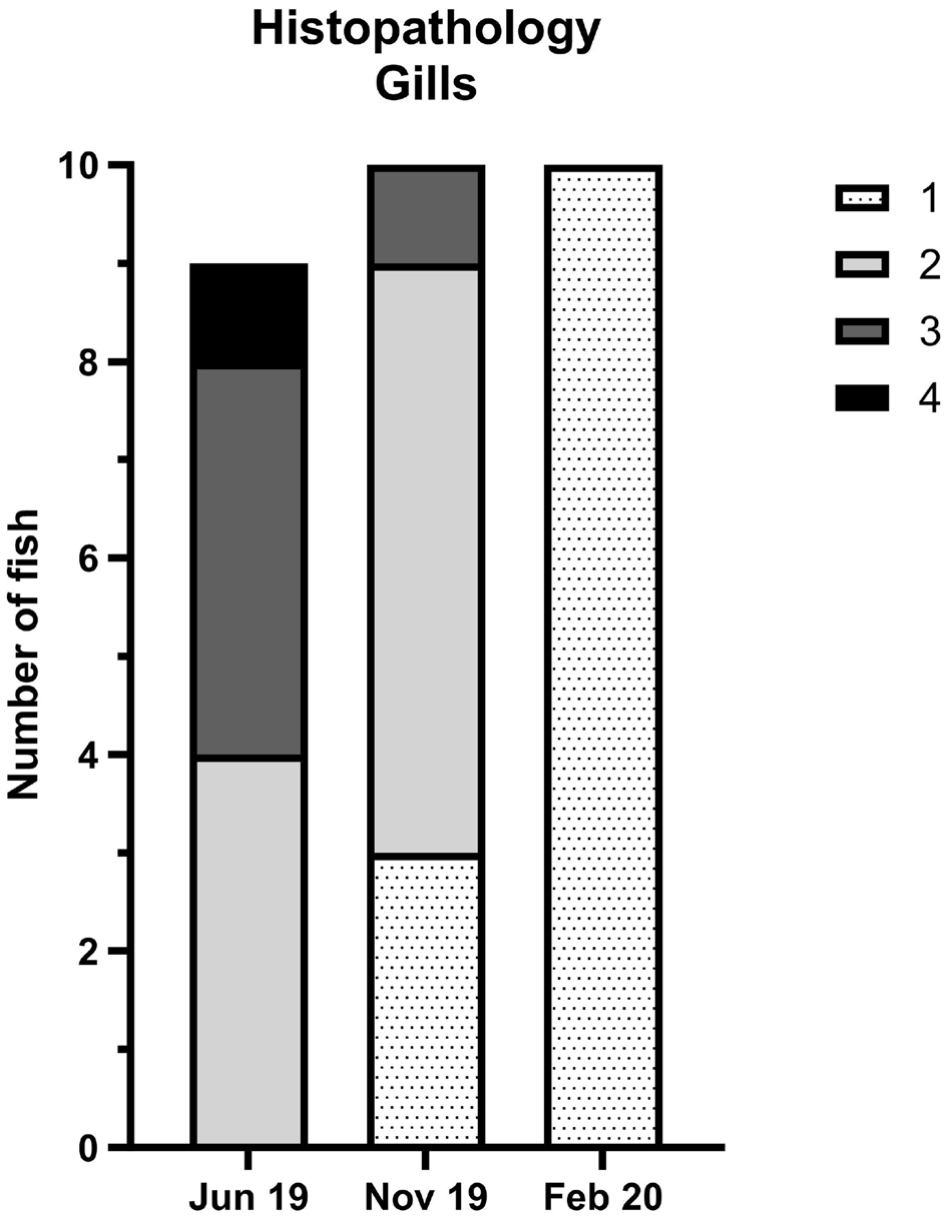
Gill histopathology of pilot study (farm A). In June 2019, only nine samples are shown (10th sample NA). Scores are given as a percentage of tissue changes in the examined slides. The percentage is a semi-quantitative estimation of how much tissue on the examined slides that no longer can be expected to contribute to the respiratory function. 1 = 1–5%, 2 = 6–25%, 3 = 26–50%, and 4 = 51–100%. The figure was generated using GraphPad Prism 10 (version 10.2.3 (403)) and Inkscape 1.2.2 (732a01da63, 2022-12-09).

In general, the first sampling at farm A (June 2019) showed the most severe pathological changes. Overall, non-specific proliferative branchitis (complex gill disorder) was seen. The lesions were mainly chronic and widespread in the gills consisting of mild clubbing of many filaments, occasionally with lymphocytic infiltration. Mixed bacteria were noted in the lumen between filaments and lamellae, but not associated to lesions. In all fish, parasites were seen in the lumen between filaments and lamellae (e.g. amoeba-like organisms and a few parasite cysts). *Ichthyophthirus* sp. was occasionally observed in the tissue associated with pathological changes. The degree of lesions in the gill was not correlated to any of the pathogens detected in the gills by standard diagnostic methods (Fig. 1). When compared to results of the 16S rRNA sequencing (Fig. 2 and Supplementary Fig. S1), the pattern of more severe lesions in the first sampling and milder lesions in the following samplings is best correlated to the detection levels of *Aeromonas*, *Flavobacterium* and *Candidatus* Branchiomonas.

### Main study: testing of high-throughput qPCR

#### Testing with synthetic DNA

First step of developing the high-throughput method for detection of multiple pathogens was to select assays. The assays for the HT-qPCR method were selected from those commonly used for detection of viral and bacterial pathogens, as well as assays previously tested and validated by HT-qPCR by Miller et al.^16^. In the case of notifiable diseases such as VHSV and IHNV, the qPCR assays listed in the diagnostic manuals were selected^17^. The HT-qPCR system chosen for this study allows for multiple singleplex qPCR reactions to occur simultaneously. Initial testing of the HT-qPCR method was performed using synthetic DNA controls (gblocks, IDT) designed specifically for each assay. The sensitivity was greatest with 18 cycles of preamplification, in which approximately 15-41 copies per µL in the starting material could be detected, with Ct values spanning from 16.81 to 31.7. Multiple assays were tested for *F. psychrophilum*, IPNV, and *R. salmoninarum*. Although some assays resulted in high Ct values during the initial testing, no assays were filtered out until further investigation using bacterial and viral isolates. Table 1 shows an overview of the copy number per synthetic DNA control and the corresponding Ct value obtained for each of the tested assays. Note that copy number refers to the input in the preamplification reaction, corresponding to initial starting material in a sample. In total, 25 assays were tested, targeting 20 different pathogens (see Supplementary Table S1).

**Table 1:** Overview of number of copies detected and Ct value at limit of detection. Copy number is known before preamplification (18 cycles).

Gblock name	Copies before PA (copies/µL)	Ct value at limit of detection (median)
ye_ruc-vi_sal-RS_MSA	27.5	RS MSA: 18.7 ye_ruc: 20.4 vi_sal: 20.9
pisck_sal-fl_psy-c_b_cys	24.3	pisck_sal: 16.8 fl_psy: 31.7 c_b_cys: 21.16
vi_ang-rlo-RS_ABC	24.2	vi_ang: 22.1 rlo: 23.1 RS ABC: 22.9
pch_sal-fly_psy_p1-ae_sal	25.6	pch_sal: 23.4 fl_psy_p1: 24.1 ae_sal: 23.4
IPNV-PRV3	15.2	IPNV SP VP2: 19.9 IPNV Tapia: 30.7 PRV-3: 18.7
Pan-PRV	25.1	Pan-PRV: 19.8
IHNV-VHSV	40.9	IHNV: 19.9 VHSV 20.4
ISAV-SAV-PMCV-PRV1	15.2	MS ISAV: 20.1 SAV: 18.3 PMCV: 17.8 PRV-1: 21.5

#### Testing with reference material

Testing was expanded using known reference material in the form of bacterial and viral isolates, and known positive tissue controls. In total, 24 assays were tested, targeting 22 different pathogens (see Supplementary Table S2). Results overview is shown in Supplementary material Table S3. Testing using bacterial and viral isolates from cultures yielded low Ct values for all tested assays, with exception of *Vibrio anguillarum* which resulted in no Ct value for both isolates. No reference material was available for *Vibrio salmonicida*, and therefore testing of that particular assay was inconclusive and further testing is required.

#### Final testing with fish tissue samples

Final testing was performed using tissue samples from experimental infection challenges with PRV-3^6^, IHNV^18^, *R. salmoninarum*^19^, *A. salmonicida*(Sepúlveda et al., in preparation), *V. anguillarum*^20^ and *F. psychrophilum*^21,22^. Results overview is shown in Supplementary material Table S4.

PRV-3 positive samples yielded a Ct value for both L1PRV3 and Pan-PRV assays, generating lower Ct values for the latter (1-1.5 Ct in difference).

*V. anguillarum* positive tissue samples yielded no Ct, thus confirming that the assay needs to be optimised.

With HT-qPCR, *R. salmoninarum* and *F. psychrophilum* generated Ct values in the relevant assays, however with some issues: *F. psychrophilum* extracted with QIAamp DNA mini kit (Qiagen) resulted in inconsistent Ct values when compared to qPCR. However, similar tissue samples extracted with NucleMag Vet (Macherey-Nagel) resulted in low Ct values (qPCR not performed). *R. salmoninarum* positive kidney samples extracted with NucleoMag Vet (Macherey-Nagel) likewise gave inconsistent results when compared to qPCR. Both of these assays require optimization, likely in the nucleic acid extraction step.

Based on the three testing steps, assays were selected for farm samples (see Table 2). Generally, one assay out of two for *R. salmoninarum, F. psychrophilum*, and one assays out of three for IPNV were discarded for each, resulting in 23 assays targeting 22 different pathogens and one assay targeting the internal amplification control (reference gene), elongation factor 1 alpha (ELF1a). The only exception was IPNV for which two assays were included. Assays for *V. anguillarum* and *V. salmonicida* were included during the farm sample testing to efficiently utilize the space on the integrated fluidic circuits (IFCs), however both assays needs to be optimized.

**Table 2:** List of qPCR assays used for farm surveillance in main study.

Target	Assay name	Sequence	Probe type	Reference
Infectious hematopoietic necrosis virus, IHNV	IHNV N	F:AGAGCCAAGGCACTGTGCG R: TTCTTTGCGGCTTGGTTGA P: AGCGGGACAGGRATGACAATGGTG	BHQ	Primers: Purcell et al.^65^ Probe: Hoferer et al.^66^
Infectious pancreatic necrosis virus, IPNV	IPNV SP VP2	F: GCCAAGATGACCCAGTCCAT R: TGACAGCTTGACCCTGGTGAT P: CCGACCGAGAACAT	MGB	Lockhart et al.^67^
	IPNV Tapia	F: GTTGATMMASTACACCGGAG R: AGGTCHCKTATGAAGGAGTC P: TACATAGGCAAAACCAAAGGAGACAC	3IABkFQ	Tapia et al.^68^
Infectious salmon anemia virus, ISAV	MS ISAV	F: CTACACAGCAGGATGCAGATGT R: CAGGATGCCGGAAGTCGAT P: CATCGTCGCTGCAGTTC	3IABkFQ	Snow et al.^69^
*Onchorhynchus masou* *herpesvirus*, OMV	OMV	F: GCCTGGACCACAATCTCAATG R: CGAGACAGTGTGGCAAGACAAC P: CCAACAGGATGGTCATTA	MGB	Lennox et al.^41^
*Piscine myocarditis* virus, PMCV	PMCV	F: TTCCAAACAATTCGAGAAGCG R: ACCTGCCATTTTCCCCTCTT P: CCGGGTAAAGTATTTGCGTC	MGB	Løvoll et al.^70^
Pan PRV (PRV-1, -2, -3)	PanPRV	F: TGGGTAACTATCAGACAAGTAACAAC R: GTAGARTCGAGTCCGCCTTCAG P: CAATTTTGGGTAACTGGCGACGGCAATGA	MGB	Zhao et al.^71^
*Piscine orthoreovirus* genotype 1, PRV-1	PRV1	F: TGCTAACACTCCAGGAGTCATTG R: TGAATCCGCTGCAGATGAGTA P: CGCCGGTAGCTCT	MGB	Palacios et al.^26^
*Piscine orthoreovirus* genotype 3, PRV-3	L1PRV3	F: TACAGGTCGTGTTCCCGTTG R: TCCAGCCACGAGGTAGATCA P: TTCAGGTTGGATGGAGCGCG	BHQ	Sørensen et al.^6^
Salmon alphavirus 1, 2, and 3, SAV	SAV QnsP1	﻿F: CCGGCCCTGAACCAGTT R: GTAGCCAAGTGGGAGAAAGCT P: CTGGCCACCACTTCGA	MGB	Hodneland and Endresen^72^
Viral hemorrhagic septicemia virus, VHSV	VHSV Spjo	F: AAACTCGCAGGATGTGTGCGTCC R: TCTGCGATCTCAGTCAGGATGAA P: TAGAGGGCCTTGGTGATCTTCTG	31ABkFQ	Jonstrup et al.^73^
Salmon gill pox virus	sgpx	F: ATCCAAAATACGGAACATAAGCAAT R: CAACGACAAGGAGATCAACGC P: CTCAGAAACTTCAAAGGA	BHQ	Gjessing﻿ et al.^24^
*Aeromonas* *salmonicida*	ae_sal	F: TAAAGCACTGTCTGTTACC R: GCTACTTCACCCTGATTGG P: ACATCAGCAGGCTTCAGAGTCACTG	MGB	Keeling et al.^74^
*Candidatus Branchiomonas* *cysticola*	c_b_cys	F: AATACATCGGAACGTGTCTAGTG R: GCCATCAGCCGCTCATGTG P: CTCGGTCCCAGGCTTTCCTCTCCCA	MGB	Mitchell et al.^14^
*Flavobacterium* *psychrophilum*	fl_psy_p1	F: GAAGATGGAGAAGGTAATTTAGTTGATATT R: CAAATAACATCTCCTTTTTCTACAACTTGA P: AAACGGGTATTCTTCTTGCTACA	MGB	Strepparava et al.^53^
*Piscichlamydia* *salmonis*	pch_sal	F: TCACCCCCAGGCTGCTT R: GAATTCCATTTCCCCCTCTTG P: CAAAACTGCTAGACTAGAGT	MGB	Nylund et al.^75^
*Piscirickettsia* *salmonis*	pisck_sal	F: TCTGGGAAGTGTGGCGATAGA R. TCCCGACCTACTCTTGTTTCATC P: TGATAGCCCCGTACACGAAACGGCATA	MGB	Corbiel et al.^76^
Red mark syndrome Rickettsia-like organism	rlo	F: GGCTCAACCCAAGAACTGCTT R: GTGCAACAGCGTCAGTGACT P: CCCAGATAACCGCCTTCGCCTCCG	BHQ	Lloyd et al.^77^
*Renibacterium* *salmoninarum*	rs_msa	F: GGAGCAACTCCGGTTACTGGTA R: TGGCCGTCCTTGAACCAT P: TGGTCTGGCGACAACAACACGTATGGT	BHQ	Bruno et al.^52^
*Vibrio anguillarum*	vi_ang	F: CCGTCATGCTATCTAGAGATGTATTTGA R: CCATACGCAGCCAAAAATCA P: TCATTTCGACGAGCGTCTTGTTCAGC	MGB	Miller^16^
*Vibrio salmonicida*	vi_sal	F: GTGTGATGACCGTTCCATATTT R: GCTATTGTCATCACTCTGTTTCTT P: TCGCTTCATGTTGTGTAATTAGGAGCGA	MGB	Miller^16^
*Yersinia ruckeri*	ye_ruck	F: TCCAGCACCAAATACGAAGG R: ACATGGCAGAACGCAGAT P: AAGGCGGTTACTTCCCGGTTCCC	BHQ	Miller et al.^78^
*Tetracapsuloides* *bryosalmonae*, PKD	te_bry	F: GCGAGATTTGTTGCATTTAAAAAG R: GCACATGCAGTGTCCAATCG P: CAAAATTGTGGAACCGTCCGACTACGA	MGB	Bettge et al.^79^
Elongation factor 1 alpha	ELF1a	F: CCCCTCCAGGATGTCTACAAA R: CACACGGCCCACGGGTACT P: ATCGGCGGTATTGGAAC	31ABkFQ	Jonstrup et al.^73^

### Main study, farm B: pathogen prevalence by standard diagnostics

**Figure 3:**
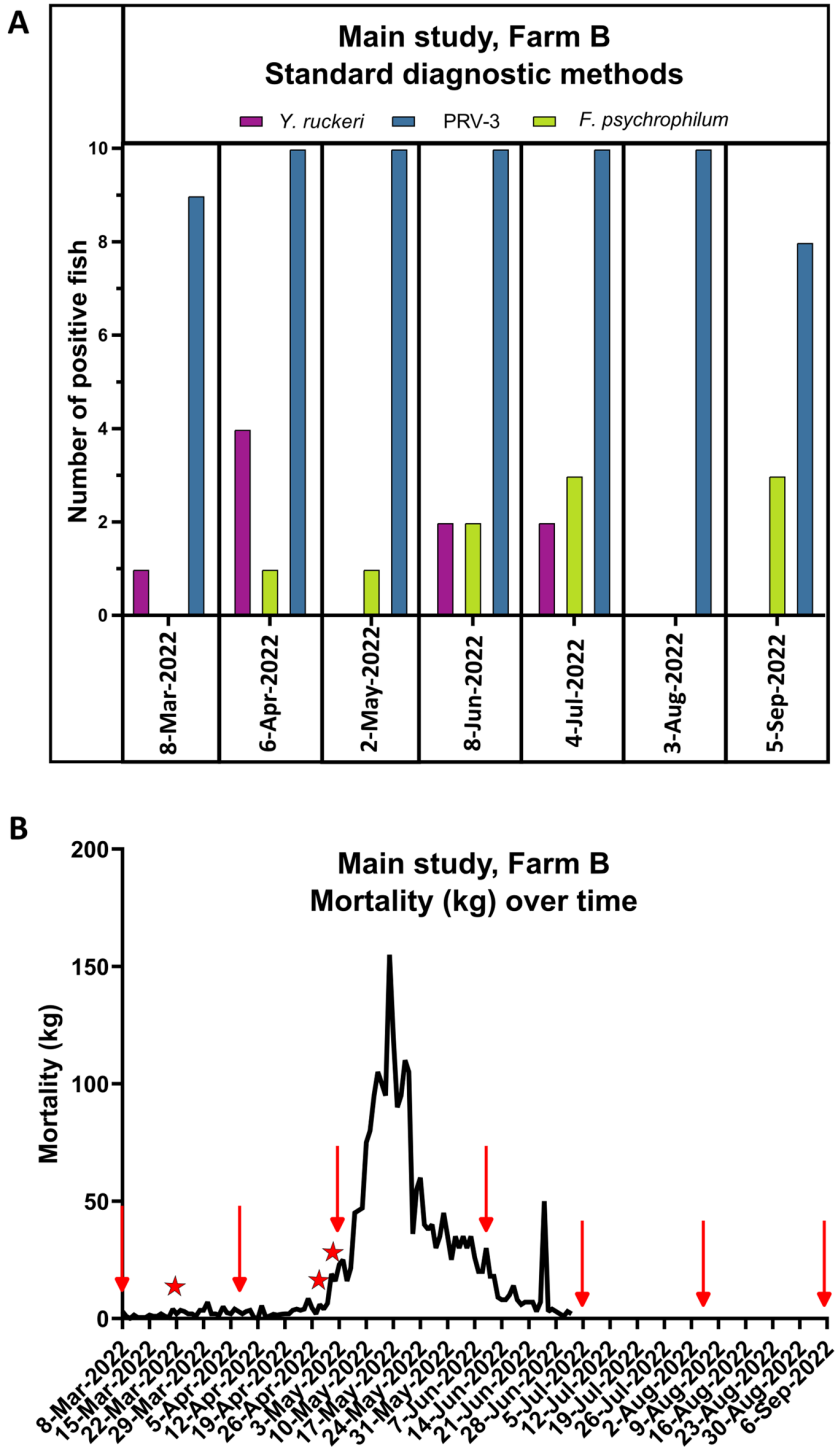
Main study, farm B. (**A**) Results by standard diagnostic methods, shown as number of fish positive for PRV-3 (blue), *F. psychrophilum* (green), and *Yersinia ruckeri* (purple). (**B**) Mortality over time. The red stars indicate (1) treatment with antibiotics, (2) moving of fish to a new unit, (3) start of increased mortality. The red arrows indicate sampling points. Mortality was not recorded after 2nd of July. The figure was generated using GraphPad Prism 10 (version 10.2.3 (403)) and Inkscape 1.2.2 (732a01da63, 2022-12-09).

Throughout the sampling period from March to September 2022 in the main study (farm B), sporadic detection (one to four fish per time point) of *F. psychrophilum* and *Y. ruckeri* were observed by standard diagnostic methods (isolation on agar and identification by MALDI-TOF^23^). PRV-3 was detected in eight to ten samples per time point in the a pool of heart, spleen and kidney (see Fig. 3), with samples above Ct 38 being considered negative. IHNV, VHSV, epizootic hematopoietic necrosis virus (EHNV), and IPNV were not detected by standard virological methods, and *R. salmoninarum* and *F. psychrophilum* were not detected by the qPCR used for routine diagnostics.

During May to mid June 2022, the farm experienced a disease outbreak in the examined cohort where mortality totalled in 2.2 tons fish (approximately 37% of the cohort). At this time point, only PRV-3 was observed in all tested individuals, and *F. psychrophilum* was detected in one out of five clinically affected fish, and none of the five clinically healthy fish.

*Yersinia ruckeri* was detected in four clinically affected fish in the beginning of April leading to treatment with antibiotic (oxolinic acid/Linacivet). The bacterium was detected again only after fish were moved to a new farming unit, and after the time period with increased mortality (June and July). Additionally, *F. psychrophilum* was observed at five out of seven time points, in a small proportion of both clinically affected and healthy fish (one to three fish per time point) (Fig. 3).

### Main study, farm B: farm surveillance with high-throughput qPCR

#### Main study, farm B: HT-qPCR on fish samples

**Figure 4:**
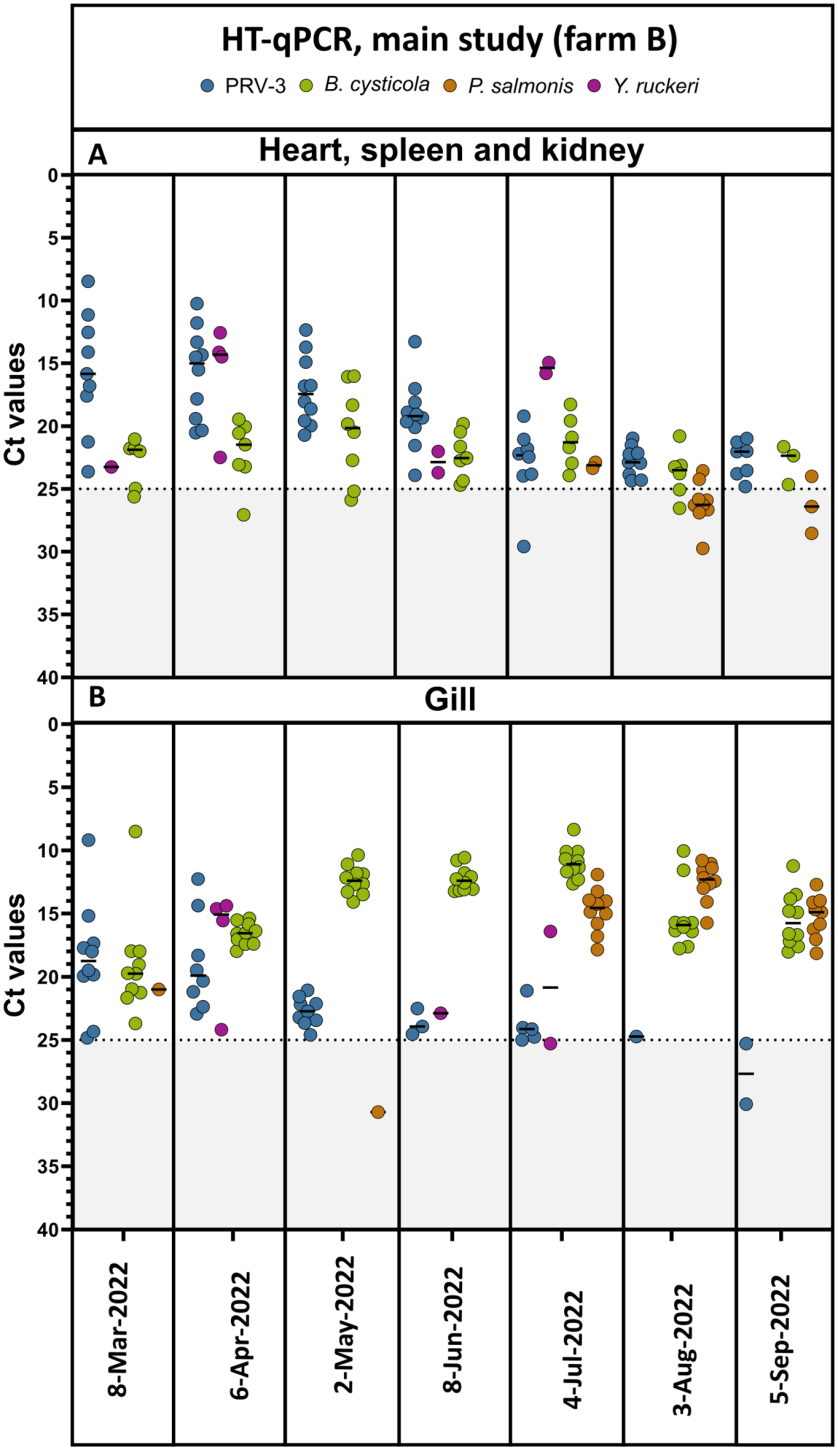
Main study, farm B. Overview of data collected by high-througput qPCR using (**A**) a pool of internal organs and (**B**) gills. Ct values per sample of PRV-3 (blue), *Candidatus* Branchiomonas cysticola (green), *Yersinia ruckeri* (purple), and *Candidatus* Piscichlamydia salmonis (orange). Horizontal line shows median value for each pathogen. Threshold for positive samples set to Ct 25. The figure was generated using GraphPad Prism 10 (version 10.2.3 (403)) and Inkscape 1.2.2 (732a01da63, 2022-12-09).

HT-qPCR allowed screening for a large number of pathogens simultaneously including the ones that are regularly tested by standard diagnostic methods. As the putative gill pathogens *Ca.* B. cysticola and *Ca.* P. salmonis had been found in the pilot study, assays for these bacteria were included in the HT-qPCR setup, allowing for description of a broader pathobiome. Both *Ca.* B. cysticola and *Ca.* P. salmonis were detected by HT-qPCR (Fig. 4). In the internal organs the prevalence of PRV-3 was 70–100%, *Ca.* B. cysticola was 30–70%, *Ca.* P. salmonis was 0–100%, and *Y. ruckeri* was 0–40% by HT-qPCR. From July to September, *Ca.* P. salmonis was detected in all examined fish, but was only found in one fish from March to June (n = 10 per month). In gills, the picture was slightly different: The prevalence of PRV-3 started at 90–100% in March to May and decreased to 20% in September, while the prevalence of *Ca.* B. cysticola was 100% at any given time point. *Ca.* P. salmonis could not be detected until July, however at 100% prevalence in July to September. Finally, *Y. ruckeri* had a low prevalence (0–40%), and was only detected in clinically affected fish.

#### Main study, Farm B: HT-qPCR on water samples

In addition to tissue samples, water was collected from the unit and filtered. Table 3 shows Pearson’s correlation analysis of pathogens found in water vs. clinically affected vs. clinically healthy individuals.

**Table 3:**
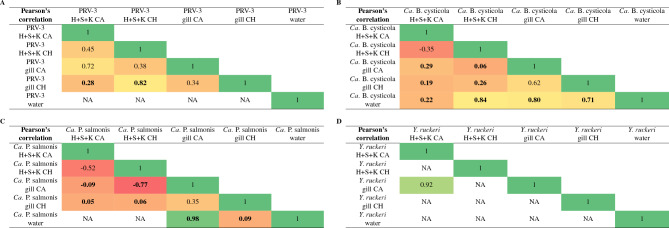
Pearson’s correlation across sampling matrixes and fish groups, comparing water vs. clinically affected (”CA”) vs. clinically healthy (”CH”) looking individuals (farm B).

When comparing fish groups, gills and internal organs (heart, spleen, and kidney (H + S + K)) are compared. (A) PRV-3, (B) *Ca.* B. cysticola, (C) *Ca.* P. salmonis, and (D) *Y. ruckeri*. Bold = p < 0.05 (t-test). NA = Most or all samples with No Ct, correlation analysis cannot be calculated.

PRV-3 was not detected in the water at any time point with the described water filtering protocol. *Yersinia ruckeri* was consistently detected in water samples throughout the study period, however it was only detected sporadically in clinically affected fish. Similarly, *F. psychrophilum* and *R. salmoninarum* were detected in water samples at multiple time points, while neither were detected by HT-qPCR in fish samples. However, *Ca.* B. cysticola and *Ca.* P. salmonis were detected in the water corresponding to the time points in which these pathogens were detected in tissue samples (Table 4, Supplementary Fig. S2). The correlation between detection in water, internal organs and gill samples of both clinically affected and healthy fish was statistically significant (p < 0.05) for *Ca.* B. cysticola. *Ca.* P. salmonis was primarily found in the gills, and a positive correlation (coefficient of 0.98) was observed in clinically affected fish, while a negative correlation was found in clinically healthy fish (coefficent of 0.09).

**Table 4:** Overview of results generated by high-throughput qPCR of samples collected during the main study (farm B).

Month	Sample type	Group	PRV-3 median Ct	No. of positive	*Ca.*. B. cysticola median Ct	No. of positive	*Ca.* P. salmonis median Ct	No. of positive	*Y. ruckeri* median Cts	No. of positive
March	H + S + K	CA	19.4	4	24.0	2	No Ct	0	23.3	1
		CH	14.1	5	21.9	5	No Ct	0	No Ct	0
	Gill	CA	19.9	2	20.9	5	21.0	1	No Ct	0
		CH	18.0	5	19.7	5	No Ct	0	No Ct	0
	Water		24.6	2 of 3*	21.7	3 of 3	29.4	1 of 3*	23.1	2 of 3*
April	H + S + K	CA	13.3	5	21.5	5	No Ct	0	14.3	4
		CH	17.8	5	23.1	4	No Ct	0	No Ct	0
	Gill	CA	18.3	5	16.5	5	No Ct	0	15.1	4
		CH	21.8	3	16.6	5	No Ct	0	No Ct	0
	Water		39.9	1 of 3*	18.6	3 of 3	28.9	2 of 3*	22.4	3 of 3
May	H + S + K	CA	16.8	5	19.8	5	No Ct	0	No Ct	0
		CH	18.6	5	25.5	4	No Ct	0	No Ct	0
	Gill	CA	23.4	5	11.9	5	No Ct	0	No Ct	0
		CH	22.2	4	13.3	5	No Ct	0	19.5	1
	Water		No Ct	0 of 3	17.7	3 of 3	29.5	1 of 3*	20.8	3 of 3
June	H + S + K	CA	18.9	5	22.6	4	No Ct	0	34.3	1
		CH	19.3	5	23.4	5	No Ct	0	30.2	1
	Gill	CA	24.4	4	12.1	5	No Ct	0	23.9	2
		CH	24.5	4	13.0	5	No Ct	0	No Ct	0
	Water		23.3	1 of 3*	17.3	3 of 3	26.1	3 of 3	21.2	3 of 3
July	H + S + K	CA	23.7	4	21.8	5	22.9	1	15.8	2
		CH	22.4	5	23.2	3	23.3	1	No Ct	0
	Gill	CA	22.9	5	10.8	5	14.0	5	16.1	2
		CH	24.4	3	16.1	5	14.9	5	No Ct	0
	Water		39.5	1 of 3*	18.1	3 of 3	16.5	3 of 3	22.1	3 of 3
August	H + S + K	CA	22.9	5	25.8	4	26.3	1	No Ct	0
		CH	22.9	5	23.2	3	26.2	1	No Ct	0
	Gill	CA	24.7	4	15.7	5	12.2	5	No Ct	0
		CH	24.5	3	16.1	5	12.9	5	No Ct	0
	Water		No Ct	0 of 3	21.4	3 of 3	15.0	3 of 3	23.8	2 of 3*
September	H + S + K	CA	22.0	4	23.1	2	23.9	1	No Ct	0
		CH	23.5	5	22.4	1	No Ct	0	No Ct	0
	Gill	CA	30.1	2	16.7	5	16.2	5	No Ct	0
		CH	25.3	1	14.8	5	14.7	5	No Ct	0
	Water		24.9	1 of 3*	18.1	3 of 3	17.7	3 of 3	22.8	3 of 3

For each of the pathogens detected by the HT-qPCR method (PRV-3, *Ca.* B. cysticola, *Ca. P. salmonis* and *Y. ruckeri*, respectively), the median Ct value is listed for a pool of heart, spleen and kidney (H + S + K), gills and water. For tissue samples (heart, spleen and kidney, and gill), number of positive refer to number of positive samples with Ct below 25. For water samples, number of positive refers to number of replicates with a Ct value (n = 3). If less than three replicates are positive, the sample is considered negative (marked with *). Additionally, Ct above 25 is considered negative. *CA* Clinically affected (n = 5), *CH* Clinically healthy (n = 5).

#### Main study, farm B: laboratory data compared with clinics

Different trends in pathogen load were observed throughout the time course study in relation to the disease outbreak. While PRV-3 levels were high at the beginning of the study and then decreased in the internal organs in relation to the increased mortality, the level of *Ca.* B. cysticola increased in the gills up to the disease outbreak, and then slightly decreased (August–September). The presence of *Ca.* P. salmonis was not observed until July, where it was present in the gills of nearly all fish at high loads (Fig. 4b), and was thus not found in association with increased mortality.

During veterinary inspection of the farm in May (at the time of increased mortality), both bacterial gill infection and *Icthyophthirius multifilis* were observed at two separate dates. While histopathology was not performed and thereby no information on gill pathology is available, the clinical observation of bacterial gill infection fits well with the high levels of *Ca.* B. cysticola in the gills detected by HT-qPCR at the same timepoint.

## Discussion

Accurate and fast pathogen detection is essential for implementing appropriate prophylactic and control measures to mitigate negative disease effects, thus enabling sustainable aquaculture production. In recent years, discovery of uncultivable microbial putative pathogens by NGS^3,24–27^ and the consequently improved diagnostic tools has highlighted the occurrence of multiple infections associated with complex disease cases both in wild populations, sea farmed and RAS farmed fish^11,15,28–32^.

In RAS, this is arguably the result of production intensification, which potentially pose an increased risk for multiple infections given the more frequent introduction of fish batches compared to traditional flow through farms and the lack of all-in-all-out strategy which is used for salmon production in net pens. Furthermore, biofilms, as a biological niche, are more frequent in RAS where they may serve as pathogen reservoirs and hamper eradication procedures^31,33^.

Infection with multiple pathogens is not limited to aquaculture, and has also been observed in wild populations. A study on wild Pacific salmon screened samples of Chinook (*Oncorhynchus tshawytscha*), coho (*Oncorhynchus kisutch*) and sockeye salmon (*Oncorhynchus nerka*) against 56 infectious agents, in order to determine their prevalence. Altogether, 41 infectious agents were detected, with individual fish testing positive for approx. 4–5 pathogens, and little variation was identified between species and the season in which the samples were collected^29^. This study also showed high prevalence of *Ca.* B. cysticola in all three species across all seasons. Another study by Deeg et al.,^34^ surveyed coho, chum (*Oncorhynchus keta*), pink (*Oncorhynchus gorbuscha*), and sockeye salmon in the Gulf of Alaska during winter, to report on the infection profile and general health status in wild populations. *Ca.* B. cysticola was found as one of the primary infectious agents with high prevalence in all species (56-89%). In addition to pathogen detection, this study examined various host markers for infection, in which both coho and sockeye salmon showed gene expression profiles correlating with *Ichthyophonus hoferi* prevalence^34^.

In this study we set out to increase the understanding of infectious disease in RAS, and to develop a tool for investigating complex diagnostic cases. This was done by a two-step approach, where we first screened the pathobiome and gill histopathology occurring in RAS farmed rainbow trout (farm A), and then designed, developed, and tested a high-throughput qPCR chip to assess pathogen load fluctuations in RAS (farm B), correlating them with disease signs.

The main findings of the pilot study were the simultaneous detection of multiple pathogens, and the presence of *Ca.* B. cysticola and *Ca.* P. salmonis, two putative emerging pathogens not previously detected in farmed rainbow trout. These bacteria have been associated with the pathobiome of farmed Atlantic salmon suffering complex gill disease (CGD)^35^. Although the detection of pathogens is not directly associated to gill pathology, the finding warrant further investigation. The pilot study relied mostly on standard diagnostic methods, however there are challenges associated with these methods for broad screenings of populations: (1) it requires different sample types depending on the analysis, (2) isolation of virus from cell culture and bacteria on agar plates rely on the presence of viable and cultivable pathogens, therefore excluding the ones for which in vitro systems are not suitable such as PRV-3^36^, (3) standard PCR assays are often single pathogen assays unless assays are multiplexed. As expected, the gill microbiome profiling performed in the pilot study (farm A) did not always comply with the findings by standard diagnostic methods. 16S rRNA analysis highlights the presence of the most common bacteria in the sample, regardless of whether they are environmental or tightly associated with gill mucosa. This could explain why, at the third sampling point (farm A, February 2020), the 16S rRNA showed an over-representation of *Rhodoferax* (84.1–90.2% of the reads), a bacterium commonly found in stagnant water and ponds with high levels of nutrients^13^, whereas standard diagnostics highlighted the presence of *F. psychrophilum*. Therefore, integrating both methods provide advantages when investigating disease in aquaculture.

The microbial pathogens detected in the pilot study along with an array of known pathogens for salmonids were included in the HT-qPCR chip design. The use of such a high-throughput tool for fish pathogen screening is not new, as it has been used for the purpose of screening populations of wild salmonids^29,34,37–41^. The advantage of including such a tool in pathogen surveillance schemes in aquaculture with repeated sampling is to decipher trends and fluctuations in relative pathogen loads, which may be predictive of increased health risks or disease outbreaks.

Initial testing of the HT-qPCR was performed step-wise, including (1) synthetic controls (gblocks, synthetic DNA), (2) reference material (in vitro reference strains), and (3) tissue samples from experimental challenges. Most assays tested against synthetic DNA controls had a sensitivity down to 15 copies/µL in the starting material before pre-amplification using 18 cycles of pre-amplification. Although, while this gives an indication of the assay sensitivity, it is uncertain if this directly applies to actual tissue samples, as the synthetic controls are DNA and the samples consist of both DNA and RNA. Additional testing was performed using reference material in the form of viral and bacterial isolates, and finally with fish tissue samples from experimental challenges. Generally, the assays performed well with the exception of *F. psychrophilum* and *V. anguillarum*. A discrepancy between qPCR and HT-qPCR targeting *F. psychrophilum* was observed in some cases. Additionally, the assay targeting *V. anguillarum* failed to detect the reference isolates and positive tissue samples. These two specific assays need to be refined. Finally, as reference material was unavailable for *V. salmonicida*, this assay needs to be validated further.

The assays were subsequently tested under field conditions, comparing with standard diagnostic methods. During the surveillance of farm B, a severe disease outbreak with increased mortality (2.2 ton of dead fish, approx. 37% mortality) occurred at the farm providing an excellent opportunity to assess the chip performances. The disease outbreak was preliminarily characterized by gill infection and observation of both the gill parasite *I. multifiliis* and bacteria in fresh smears. The results from traditional laboratory diagnostic showed that PRV-3 was prevalent at all time points (tested by qPCR in pooled organ material) whereas sporadic detection of fish pathogenic bacteria *Y. ruckeri* and *F. psychrophilum* (kidney swabs) occurred during the surveillance. These results did not fully explain the severity of the disease outbreak in the scrutinized fish cohort, considering that PRV-3 does not cause mortality during controlled experimental challenge^4,6^.

The HT-qPCR chip provided valuable additional information (Fig. 4), therefore allowing for increased resolution of the disease outbreak. Besides detecting PRV-3 at all time points in internal organs and in gills as well was sporadic detection of *Y. ruckeri*, we could detect two uncultivable putative gill pathogens. *Ca.* B. cysticola was highly prevalent in all gill samples collected in this study, but *Ca.* P. salmonis was only detected in the late phase of the surveillance after the mortality event. These bacteria, and in particular *Ca.* B. cysticola, have been detected in Atlantic salmon with complex gill disease, however causative relationship has not been fully established^14,15,28,42^. Gjessing et al. found high prevalence of *Ca.* B. cysticola in three farms challenged with complex gill infections. Severe gill pathology was observed in association with this bacterium^15^, contrary to a previous study^43^. The inflammatory changes Gjessing et al. observed appeared to be specifically linked to *Ca.* B. cysticola, as the lesions were different to those typically observed in relation to *Ca.* P. salmonis infections^15^. The presence of these putative gill pathogenic bacteria together with *I. multifiliis*, complies with the clinics observed in the current study, where severe gill issues were reported during the period of increased mortality, and at this time point *Ca.* B. cysticola load observed was high in the gills (May–June, Fig. 4). However, gill histopathology was not performed during the main study (farm B), and thus further data to support impairment of the gills during the disease outbreak is lacking.

The third sampling, at the onset of severe increased mortality, showed an interesting pattern in pathogen kinetics as depicted by HT-qPCR. PRV-3 load peaked in the internal organs at the sampling time prior to the disease outbreak and then decreased. This is in compliance with what was observed under experimental conditions; here, clinics caused by PRV-3 are observed during and after the peak of virus load, with hematocrit reduction occurring during the peak, and heart pathology peaking two weeks later^6,7,44^. Conversely, the load of *Ca.* B. cysticola in the gills observed by HT-qPCR increased markedly. We therefore hypothesize that the reduced capability of oxygen transportation due to PRV-3 infection is exacerbated by the gill infection with *Ca.* B. cysticola which limits exchange of oxygen from water into fish red blood cells. Future studies including gill histopathology would need to be conducted to support this theory. Furthermore, at peak of infection (PRV-3 and *Ca.* B. cysticola), the fish batch was handled and moved, thus increasing stress levels and mortalities.

The capability of testing a large sample number allowed comparison between clinically healthy and affected fish. With particular reference to the bacterial pathogens *Y. ruckeri* and *F. psychrophilum*, our data show that selection of clinically affected fish is necessary for increased diagnostic sensitivity. Conversely, PRV-3 and *Ca.* B. cysticola were equally detected in both groups, suggesting that they are more prevalent at farm level. The capability of detecting pathogens is also tissue dependent as shown by the two putative gill pathogens (*Ca.* B. cysticola and *Ca.* P. salmonis) which were consistently more prevalent in gill than in internal organs. These data suggest that in order to have a high resolution picture of the pathobiome affecting farmed fish, internal organs and gill tissue, the latter representing the interface with the farming environment, should be analyzed separately.

Herein, we also compared detection of pathogens in water versus tissue samples. Environmental DNA/RNA testing would simplify RAS sampling, but our data suggests that the diagnostic resolution is lost in water: PRV-3 could not be detected in the water, while the facultative pathogens *R. salmoninarum*, *F. psychrophilum* and *Y. ruckeri* were detected repeatedly without significant load fluctuation (data not shown for *R. salmoninarum* and *F. psychrophilum*). Pearson’s correlation analysis revealed a moderate correlation between *Ca.* B. cysticola presence in water and tissue samples, and no correlation for *Ca.* P. salmonis between water samples and infected but clinically healthy fish (see Table 3). The fact that PRV-3 could not be detected in water samples could be method specific and needs to be further investigated under experimental conditions. Additionally, it may suggest that the main transmission route of PRV-3 is through fish to fish contact and only partly mediated through water. Other studies examining pathogens in water versus fish have found similar discrepancies: a study by Hu et al. found that *Aeromonas veronii* and *Aeromonas hydrophila* were commonly isolated from diseased fish, while *A. veronii* was isolated from filtered water samples and in healthy fish. Furthermore, they found that virulence genes were primarily found in *A. hydrophila*^45^. A study by Miller et al. using zebrafish indicated that certain pathogens (*Pseudoloma neurophilia* and *Myxidium strisingeri*) were primarily found in fish and not in water^46^. Interestingly, the results from the main study (farm B) suggests that there are at least three different compartments (1. water and biofilm, 2. fish mucosa as interface (e.g. gills), and 3. internal organs) to be investigated separately in a RAS farm with regards to fish health, and the permeability of these three compartments may not be equal for all microbial agents^47^. The data collected here supports the recommendations by World Organization for Animal Health (WOAH)^48^, stating that eDNA cannot be used to declare freedom from or confirm suspicion of pathogen presence without confirmatory analysis by standardized tissue sampling. Additionally, eDNA sampling is not appropriate for surveillance purposes, as pathogens in water samples may either be a result of contamination by e.g. inactivated pathogen from heat-treated products, and therefore may not indicate infection of the host. This is demonstrated by HT-qPCR data, as e.g. *Y. ruckeri* was detected consistently in water samples, but only occasionally in a few clinically affected fish.

Another use of this HT-qPCR tool is to evaluate the effectiveness of disease control measures. The examined fish cohort was treated for an *Y. ruckeri* infection to mitigate a disease outbreak. The bacterium was only detected again after the fish had been moved to a new unit, indicating that treatment was successful at first, but the fish were exposed to a second infection with the same pathogen later. Hence, in depth pathogen surveillance can be used to evaluate the efficacy of a treatment over time. Overall, this tool, once integrated in a sampling scheme, has the potential to be a farm management tool. It will allow monitoring the pathogen load in a fish batch and consequently adjust production plans to reduce the risk of disease outbreaks by providing adequate timing to implement farming practices.

The high-throughput qPCR tool developed has currently some limitations which needs to be addressed. Failure to detect *F. psychrophilum* in tissue samples by HT-qPCR indicates a possible issue with the nucleic acid purification procedure, particularly as this bacterium was found in samples by standard diagnostic methods, and as the HT-qPCR method detected the bacterium in the water samples. The molecular assay does not discriminate between viable pathogens and remnants of nucleic acids, hence results needs to be interpreted based on clinical observations and pathogen load fluctuation in the sample time-series.

While improvements are necessary in order to fully implement the high-throughput qPCR tool for broader diagnostics and surveillance of pathogens in RAS, this study shows that multi-pathogen screening should be considered in order to support fish health and disease control strategies.

## Methods

### Pilot study

During the pilot study, samples were collected according to Table 5 in June 2019, November 2019, and February 2020 from a RAS farm. The purpose of the sampling was to screen for a panel of known pathogens by current standard diagnostic methods, and to investigate the gill microbiome by 16S rRNA sequencing.

**Table 5:** Sampling plan for pathogen investigation for pilot study (farm A).

Tissue	Type	Sampling pooling	Purpose
Kidney	Swab	Individual (30 fish)	Bacteriology
	RNAlater	Individual (30 fish)	R. salmoninarum qPCR
Heart, spleen, kidney	EMEM	Pool 1: Fish 1–10 Pool 2: Fish 11–20 Pool 3: Fish 21–30	Virology
Heart, spleen	RNAlater	Pool 1: Fish 1–5 Pool 2: Fish 6–10 Pool 3: Fish 11–15 Pool 4: Fish 16–20 Pool 5: Fish 21–25 Pool 6: Fish 26–30	PRV-3 RT-qPCR
Gills	Sterile tube	Pool 1: Fish 1–10 Pool 2: Fish 11–20 Pool 3: Fish 21–30	Microbiome, NGS
	Swab	Individual (fish 1–10)	Bacteriology
	Sterile tube	Pool 1: Fish 1–5 Pool 2: Fish 6–10 Pool 3: Fish 11–15 Pool 4: Fish 16–20 Pool 5: Fish 21–25 Pool 6: Fish 26–30	ELISA
	10% buffered formalin	Individual (fish 1–10)	Histology

#### Standard bacteriology

Kidney swabs were collected from all fish, and plated on Trypton yeast extract salts (TYES)^49^ and blood agar plates for detection of bacteria, including *Yersinia ruckeri, Flavobacterium psychrophilum, Aeromonas salmonicida*, and *Vibrio anguillarum*. Blood agar plates were incubated at 20 °C and TYES-agar plates at 15 °C for up to two weeks. Bacterial colonies were identified by MALDI-TOF (Bruker)^23^.

#### Standard virology

From each sampling event, two pools of internal organs, representing clinically affected and clinically healthy looking fish were collected in EMEM (Eagle’s Minimum Essential Medium, SSI Diagnostika) for viral isolation on cell culture. The pools contained organs from 5 specimens of heart, spleen, and kidney. These samples were processed following the diagnostic manual v2021.2 provided by the European Union Reference Laboratory (EURL) for fish and shellfish diseases. Briefly, following homogenization by mortar and pistile with sterile sand, each pooled sample was suspended 1:10 in EMEM, supplemented with 10% v/v fetal calf serum (FCS) and 2% v/v of antibiotic-antimycotic solution (Penicillin 100 UI/ml, Streptomycin sulphate 10 mg/mL, Amphotericin B 25 µg/ mL and Kanamycin 10 mg/mL) (Sigma Aldrich, USA). Tissue extracts were centrifuged at 3000×*g* for 30 min and incubated over-night at 4 °C. Samples were inoculated on 24 hour old monolayer of Bluegill fry (BF-2)^50^ and epithelioma papulosum cyprini (EPC)^51^ cell lines. Tissue extracts were inoculated at two tenfold dilutions (1:10 and 1:100) onto 1-day old, BF-2 and EPC cells grown in 24-well cell culture plates and incubated at 15 °C. After inoculation, plates were observed daily for detection of cytopathic effect (cpe). Samples were examined along one progressive passage of 7 days.

#### qPCR

Nucleic acids from samples were extracted using IndiMag Pathogens kit using IndiMag48 (IndiCal Bioscience) according to manufacturer’s recommendations.

PRV-3 detection was performed using TaqPath 1-Step Master Mix (Applied Biosystems) according to the manufacturer’s recommendations with the assay described in Sørensen et al. 2023^6^, using 5 µL RNA template in a total volume of 25 µL.

Detection of *R. salmoninarum* and *F. psychrophilum* was performed using Luna Universal qPCR Master Mix (New England Biolabs) according to manufacturer’s recommendations, using the qPCR assays described in Bruno et al.^52^ and Strepparava et al.^53^, respectively.

The positive sample cut-off was set at Ct 38.

#### Gill histopathology

Tissue samples collected in 10% neutral-buffered formalin were processed conventionally with a 13 h overnight program in an automated tissue processor (Epredia Excelsior TM), embedded in paraffin and sections of 3–4 µm were cut and stained with hematoxylin and eosin (HE). The slides were assessed in a blinded fashion by one investigator using an upright light microscope (Leica DMRB 2000 TM with 2,5 ×,10 ×, 20 ×, 40 ×, and 63 × objectives. Pathological gill changes were noted.

#### NGS of gill microbiome

In addition to the samples collected for standard diagnostic methods, gill samples were collected in June and November 2019 and February 2020. In total, 30 fish were sampled per time point, and gills were collected in 1 mL RNA later (Invitrogen) and analysed in pools of 10 fish. DNA from gill pools were extracted using DNA mini kit (Qiagen), according to manufacturer’s recommendations. 16S rRNA gene sequencing was performed by DNAsense ApS (Aalborg, Denmark). Briefly, 16S rRNA gene region V3-4 sequencing libraries were prepared using a custom protocol based on an Illumina protocol. In total, 15 ng of the extracted DNA was used as a template for PCR amplification of the specified region. The total reaction volume was 25 µL, containing 12.5 µL PCRBIO Ultra mix (PCR Biosystems) and 400 nM of both forward and reverse tailed primer mix (Forward: CCTACGGGNGGCWGCAG, reverse: GACTACHVGGGTATCTAATCC^54^). PCR was performed in duplicates per sample, which were subsequently pooled. Tailed primers were used to allow for attachment of Illumina Nextera adaptors, which are necessary for sequencing in the subsequent PCR. The amplicons were purified using Agencourt Ampure CP Beads (Beckman Coulter) with a bead to sample ratio of 4:5, and DNA was eluted in 25 µL nuclease free water (Qiagen). The DNA concentration was measured using Qubit dsDNA HS Assay kit (Invitrogen), and the product size and purity was validated using gel electrophoresis with Tapestation 2200 abd D1000/High-sensitivity D100 screentapes (Agilent) on a subset of the libraries. Library preparation was performed in 25 µL reactions using PCRBIO HiFi Polymerase (1 U/reaction) and PCRBIO HiFi Buffer (1x) (PCRBiosystems), adaptor mix (400 nM of both forward and reverse) and up to 10 ng template, and PCR was conducted using the following program: 95 °C for 2 min, (95 °C for 20 s, 55 °C for 30 s, and 72 °C for 60 sec) x 8 cycles, and 72 °C for 5 min. Libraries were purified using Agencourt Ampure XP Beads (Beckman Coulter) as previously described. DNA concentration was measured using Qubit dsDNA HS Assay kit (Invitrogen), and validation of product size and purity performed as previously described.

Sequencing was performed with MiSeq (Illumina) using MiSeq Reagent kit v3 according to manufacturer’s recommendations.

The bioinformatic analysis was conducted by DNAsense using their well established pipeline. Forward and reverse reads were trimmed using Trimmomatic v. 0.32^55^, with SLIDINGWINDOW:5:3 and MENLEN:275. Trimmed reads were merged using FLASH v. 1.2.7^56^ with -m 10 -M 250, and dereplicated for formatted for use in the UPARSE workflow^57^. Dereplicated reads were clustered using usearch v. 7.0.1090 -cluster_outs command with default setting. OTU abundances were subsequently estimated using -usearch_global command with -id 0.97 .maxaccepts 0 -maxrejects 0. Taxanomy was assigned using RDP classifier^58^ implemented in the parallel_assign_taxanomy_rdp.py script in QIIME^59^, with -confidence 0.8 and the SILVA database, release 132^60^. The results were analysed in R v. 4.0.2 using Rstudio IDE with the ampvis package v.2.6.5^61^.

Data processing was performed using DNAsense application (https://dnasense.shinyapps.io/dnasense/).

### Main study: development of high-throughput qPCR platform

#### Initial validation with synthetic controls

Initial testing and validation of the HT-qPCR method was performed using synthetic DNA controls (gblocks, Integrated DNA Technologies) specifically designed for each assay (see Supplementary Table S5 for sequences). Each articifical control was diluted to a working solution at 1E8 copies/µL, and a serial dilution from 1E7 to 1E0 copies/µL was tested in triplicates. Briefly, the diluted synthetic controls (1E7 to 1E0 copies/µL) were preamplified using AgPath-ID 1-Step RT-PCR (Agilent) at 14, 16, and 18 cycles in order to determine the appropriate protocol to reach a similar sensitivity to current qPCR methods. All samples were preamplified using a 200 nM primer mix consisting of primers from all assays listed in Tabel﻿ S1, using the following thermo profile: 20 min × 45 °C, 10 min × 95 °C, (15 s × 95 °C, 60 s × 60 °C) × 14, 16, or 18 cycles, 4 °C until end. Preamplified samples were diluted 1:5 (20 µL low EDTA TE-buffer + 5 µL sample) and stored at – 20 °C until use.

HT-qPCR was performed using 48.48 Dynamic Array (DA) Integrated Fluidic Circuit (IFC) (Standard BioTools, formerly Fluidigm), in which 48 samples are tested against 48 assays simultaneously. Briefly, primer and probe mixes of 9 µM of each primer and 2 µM of the probe were prepared, resulting in a final concentration of 4.5 µM and 1 µM of the primers and probe, respectively. qPCR was performed according to manufacturer’s recommendations using Controller MX and BioMark HD (Standard BioTools).

Data was analysed using Fluidigm Real-Time PCR Analysis (version 4.7.1, build 20200930.1707, Standard BioTools).

#### Laboratory validation with reference standards

Secondly, validation was performed using reference standards, consisting of bacterial cultures from recent diagnostic cases and virus isolates (see Supplementary Table S3). Nucleic acids were purified using MagMax Micriobiome Ultra Nucleic Acid Isolation kit (Thermo Fisher) and MagMax MirVana Total RNA Isolation kit (Thermo Fisher) for DNA and RNA isolates, respectively, following manufacturer’s recommendations.

Preamplification was performed as previously described, with 18 cycles. HT-qPCR was performed as described above with assays listed in Table S2.

#### Clinical validation with known reference samples from experimental challenges

Validation for diagnostic purpose of the HT-qPCR chip was performed using tissue samples from experimental infection challenges; PRV-3^6^, IHNV^62^, *R. salmoninarum*^63^, *F. psychrophilum*^21,22^, *Aeromonas salmonicida* (Sepúlveda et al. in preparation), and *Vibrio anguillarum*^20^.

The panel of selected samples consisted of tissues from infectious studies of other research projects conducted at the experimental fish tank facilities at DTU AQUA Kgs, Lyngby under license number 2019-15-0201-00159, and the experimental protocols were approved by the Danish Animal Research Authority. Six samples from each experimental infection challenge with varying Ct values by qPCR were chosen: heart from PRV-3, kidney from *R. salmoninarum*, heart and spleen from *F. psychrophilum*, and heart and spleen from IHNV, totalling in 24 samples.

Rainbow trout were obtained from eyed eggs provided by a Danish commercial fish farm, registered officially free of infectious pancreatic necrosis virus (IPNV), infectious hematopoietic necrosis virus (IHNV), viral hemorrhagic septicemia virus (VHSV), and *Renibacterium salmoninarum* (bacterial kidney disease, BKD). After disinfection procedures using iodine, the eggs were hatched and grown in the wet laboratory facilities of section for fish and shellfish diseases, DTU Aqua, Kgs. Lyngby, Denmark, in recirculating and UV disinfected tap water (12 °C). True positive samples were selected based on positive testing with pathogen specific gold standard methods, either qPCR (PRV-3), re-isolation on EPC cells (IHNV), re-isolation on agar (*F. psychrophilum* and *R. salmoninarum*).

Nucleic acid purification was performed using IndiMag Pathogens (Indical Biosciences) for PRV-3, IHNV, and *R. salmoninarum* and DNA Mini kit (Qiagen) for *F. psychrophilum* according to manufacturer’s recommendation.

Briefly, PRV-3 positive tissue samples were homogenized in 600 µL PBS with a 5 mm stainless steel bead (Qiagen) for 2 min at 25 Hz on TissueLyzer II (Qiagen). The samples were centrifuged at 14,000×*g* for 5 min at 4 °C, and total RNA was extracted from 200 µL supernatant using the IndiMag Pathogen kit (Indical Biosciences). IHNV samples were processed with mortar and pestle, and centrifuged at 4 °C at 4000×*g*. RNA was extracted using IndiMag Pathogen kit (Indical Biosciences). R. salmoninarum positive tissue samples were extracted using NucleoMag Vet kit as previously described.

Finally, DNA from *F. psychrophilum* positive tissue samples was purified using DNA mini kit (Qiagen) according to manufacturer’s recommendations. Briefly, tissue was transferred to a new 2 ml tube with a 5 mm stainless steel bead (Qiagen) and 80 µL of PBS and lyzed with a TissueLyzer II (2 min at 25 Hz). 100 µL of ATL buffer plus 20 µL of proteinase K were added, and samples were incubated at 56 °C overnight. Subsequently, the manufacturer’s protocol was followed (elution in 100 µL).

Preamplification was performed as previously described, with 18 cycles. HT-qPCR was performed as described above.

### Main study: surveillance of RAS farm and field validation of HT-qPCR

#### Study design

From March to September 2022, monthly sampling was performed from ten fish from the same batch, selecting five clinically healthy and five clinically affected fish. Table 6 lists the samples collected at each time point and the intended use. Additionally, production data (data not shown) was recorded during the seven months, including data on weight, feeding, disease outbreaks, treatments, and water quality parameters.

**Table 6:** Samples collected at each time point for the duration of the field sampling of farm B (main study).

Tissue	Type	Purpose
Heart, spleen, kidney	RNAlater	RT-qPCR and high-throughput qPCR
Gill	RNAlater	High-throughput qPCR
Kidney	Swab	Bacteriological examination
Heart, spleen, kidney	Medium	Viological examination
Water (2L)		High-throughput qPCR

#### Bacteriology and virology

Standard bacteriology and virology was performed as previously described for the pilot study.

#### qPCR

Nucleic acids were extracted from pooled heart, spleen and kidney material with NucleoMag Vet (Macherey-Nagel) using Kingfisher Flex (Thermo Fisher) according to manufacturer’s recommendations. Briefly, approximately 25mg tissue was homogenized in 600 µL Buffer RA1 (Macherey-Nagel) with a 5 mm stainless steel bead (Qiagen) and zirconia beads (Thermo Fisher) using TissueLyser II (Qiagen) at 25 Hz for 4 minutes. After homogenization, samples were centrifuged at 14,000×*g* for 2 mins at 4 °C (Ole Dich). 200 µL of the supernatant was used for purification. Nucleic acids were stored at – 80 °C after purification. qPCR was performed as previously described in the pilot study.

#### Sample processing and high-throughput qPCR of field samples

Water testing was done according to the method by Zarantonello and Cuenca^64^. Briefly, 2 L of water were collected at each time point from the same location and depth of the selected unit. Water samples were filtered within 24 hours of receipt (typical shipment time was approx. 24 h at 4 °C), using bottle-top filtration system (Nalgene) connected to a vacuum pump. The water was filtered through PVDF membranes of 47 mm in diameter with a pore size of 0.22 µm (EMD Milipore Durapore). All samples were filtered in triplicates, passing approximately 100–400 mL water through each filter until clogging occurred. Filters were folded and cut to fit in 2 mL sterile tubes, and immediately snap frozen on dry ice.

Before DNA/RNA purification, filters were processed using TissueLyser II as previously described, but using only zirconia beads (Thermo Fisher). Nucleic acid purification for tissue samples and filters were performed as previously described using NucleoMag Vet kit (Macherey-Nagel). All samples were preamplified as previously described with 18 cycles of preamplification in duplicates, and diluted 1:5 in low EDTA TE-Buffer (AppliChem GmbH), and stored at – 20 °C until further use. HT-qPCR was performed as previously described according to manufacturer’s recommendations using assays from Table 2 with a final primer and probe concentration of 4.5 µM and 1 µM, respectively, using 192.24 dynamic array with controller RX and BioMark HD.

### Ethics declaration

Samples used in the laboratory testing of the HT-qPCR assay originated from studies published elsewhere as described previously. All the animal studies referred to were reviewed and approved by the Animal Experiments Inspectorate (Ministry of Food, Agriculture and Fisheries of Denmark), under the license to conduct in vivo animal studies number 2019-15-0201-00159. Samples collected from fish farms were part of diagnostic investigation and, hence, out of the scope of the regulations for animal studies (DIRECTIVE 2010/63/EU).

## Supplementary Information

Supplementary Information.

## Data Availability

The gill microbiome dataset generated during the current study are available in the Sequence Read Archive (SRA, NCBI) repository, BioProject accession number PRJNA1049333. The remaining data are available from the corresponding author on reasonable request.
