# Correction: A novel high-throughput qPCR chip for solving co-infections in RAS farmed rainbow trout

**DOI:** 10.1038/s41598-026-36370-z

**Published:** 2026-01-28

**Authors:** Juliane Sørensen, Argelia Cuenca, Jacob Günther Schmidt, Simon Brøndgaard Madsen, Tine Moesgaard Iburg, Lone Madsen, Niccoló Vendramin

**Affiliations:** 1 National Institute of Aquatic Resources DTU Aqua, Section for Fish and Shellfish Diseases, Technical University of Denmark, Kgs. Lyngby, 2800 Denmark; 2 Højslev, 7840 Denmark

Correction to: *Scientific Reports* 10.1038/s41598-024-65697-8, published online 22 July 2024

The original version of this Article contained errors in Table 2, where the R and P sequences for Target “*Piscine orthoreovirus* genotype 1, PRV-1”, and the P sequence for Target “Salmon alphavirus 1, 2, and 3, SAV” were incorrect. The correct and incorrect values appear below.

Incorrect:

**Table:** 

Target	Sequence
*Piscine orthoreovirus* genotype 1, PRV-1	R: ACCTGCCATTTTCCCCTCTT P: CGCCGGTAGCTCC
Salmon alphavirus 1, 2, and 3, SAV	P: TCGAAGTGGTGGCCAG

Correct:

**Table:** 

Target	Sequence
*Piscine orthoreovirus* genotype 1, PRV-1	R: TGAATCCGCTGCAGATGAGTA P: CGCCGGTAGCTCT
Salmon alphavirus 1, 2, and 3, SAV	P: CTGGCCACCACTTCGA

In addition, Reference 74 contained an error and was incorrectly stated as:

Keeling, S. E. *et al.* Development and validation of real-time PCR for the detection of *Yersinia ruckeri*: *Yersinia ruckeri* real-time PCR. *J. Fish Dis.* **35**, 119–125. 10.1111/j.1365-2761.2011.01327.x (2012).

The correct reference is listed below:

Keeling, S.E. *et al.* Development and validation of a real-time PCR assay for the detection of *Aeromonas salmonicida*, *J. Fish Dis.*, **36**(5), 495–503. 10.1111/jfd.12014 (2013).

The original Article has been corrected.

